# Supplementary figures and images for: Impact of local mask mandates upon COVID-19 case rates in Oklahoma
Source: PLoS One. 2022 Jun 16;17(6):e0269339. doi: 10.1371/journal.pone.0269339 (PMC9202880; doi:10.1371/journal.pone.0269339)

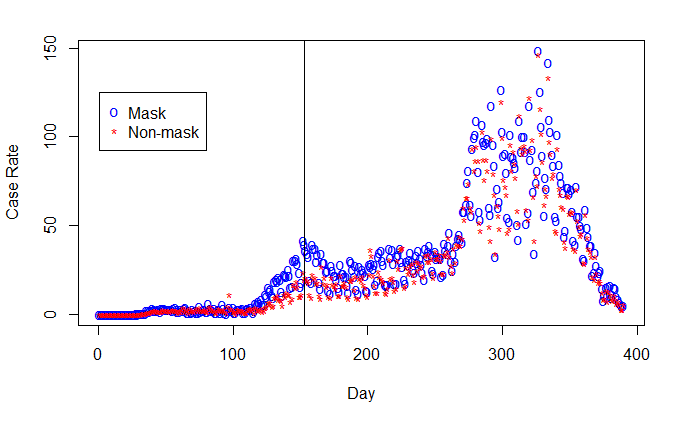

Supplement: S1 Fig — (JPG) [file pone.0269339.s001.jpg]
