# Supplementary material for: Impact of local mask mandates upon COVID-19 case rates in Oklahoma
Source: PLoS One. 2022 Jun 16;17(6):e0269339. doi: 10.1371/journal.pone.0269339 (PMC9202880; doi:10.1371/journal.pone.0269339)
Supplement: Attachment — Submitted filename: response to reviewers Apr.docx [file pone.0269339.s005.docx]

Response to editor:

Comment was made in the review regarding citation of retracted articles. I am unclear if this is a standard request or a specific citation raised concern. All citations were verified as having no retractions. One article had an erratum issued in a subsequent publication. Information on this has been added to the references section. This addition is the only change made to the manuscript itself. The only other changes requested were in relation to figures 1 and 3.

Response to reviewers:

The authors are again appreciative of the time and expertise of the reviewers. No comments were provided from reviewer #1 for the resubmission, and thus no actions were taken. Reviewer #2 provided suggestions for figure #1, many of which were adopted. It is the authors’ opinion that other recommendations (specifically, changing the reference of dates to days) would misrepresent the data analysis process. For Step 2, the reviewer asked: “Is this number of cases for days or dates? If days, then consider revising to ‘…..each day (d-45 to d90)…..” The number at that step is for specific dates. The conversion to days does not occur until after the difference has been calculated for each municipality (i.e., rate for municipality - rate in remainder of the state), which is shown as step 8. The data analysis had to be done in this order was because there is no single “d0” rate for the non-mandated portion of the state. Conceptually, the figure could be altered to state that the differences were calculated for each day count without necessarily changing the interpretation. This would indeed simplify the figure. However, since this is not consistent with the actual method of data analysis, the authors are reluctant to adopt such changes.

Figure 3 was updated with truncation of data to be consistent with intervals of the study period.
