# Supplementary material for: Impact of local mask mandates upon COVID-19 case rates in Oklahoma
Source: PLoS One. 2022 Jun 16;17(6):e0269339. doi: 10.1371/journal.pone.0269339 (PMC9202880; doi:10.1371/journal.pone.0269339)
Supplement: Attachment — Submitted filename: PONE-D-21-39472 Reviewer Comments.docx [file pone.0269339.s003.docx]

**Overall**

This article makes a valid contribution to the scientific record by evaluating the efficacy of local mask mandates in reducing COVID-19 case rates. The present work highlights an important limitation of previous mask mandate efficacy research and employs an analytic strategy that minimizes this limitation. Previous research has focused on comparing individual municipalities with themselves at two or more different points in time (i.e., prior to and following mask mandate implementation). These strategies are limited by their inability to largely account for temporal changes in other factors besides mask mandates that may influence COVID-19 transmission. To address this longitudinal limitation, the present manuscript instead compares mandated and non-mandated municipalities in Oklahoma at the same points in time. Specifically, a modified difference-in-difference analytic approach was used to compare the difference in case rates between 40 Oklahoma municipalities that eventually implemented a mask mandate and the rest of the non-mandated municipalities at the same points in time. The finding that locally-mandated mask mandates can mitigate differences between these two groups even when COVID-19 case rates are initially higher in the “eventual mandate” population than the “never mandate” population prior to implementation is very encouraging.

While this work makes a substantive contribution to knowledge, some minor revisions are first required to improve readability, resolve conflicting or confusing statements, add a little bit of information to the Methods and Materials, fix in-text and Table errors, and explain/elaborate on certain statements.

**Data Availability**

I know the authors can’t share raw data owing to the protection of confidential medical information. However, the authors could provide the case rate for each municipality and non-mandate population at each d(-45) to d(90) time point.

**Abstract**

**Lines 28-31:**

In the abstract, the slope of difference estimates given in brackets may be difficult for the reader to understand since they have not yet read the paper. Word count permitting, I’d like to suggest the authors explain the first slope of difference in words. For example:

“Prior to adopting mask mandates, those municipalities that eventually adopted mandates had higher transmission rates than the rest of the state, with the mean case rate difference per 100,000 people increasing by 0.32 per unit increase in time (slope of difference= 0.32; 95% CI 0.13 to 29 0.51).”

If the authors cannot do this in the abstract, I’d recommend doing something similar in the results the first time an estimate for the slope of mean case rate difference is presented.

**Introduction**

The authors provide an excellent summary of the COVID-19 timeline and mitigation efforts, a thorough background of existing research, a clear identification of research gaps, and a sound justification for the present work. However, the authors should consider reorganizing the summary of existing research to enhance reader clarity.

**Line 48-49:**

Could the authors briefly list some of the other “number of factors” besides “limited adoption” in this sentence (e.g., politicization of masks, economic burden, etc.)?

**Lines 51-104 (Paragraph 2):**

This is a very long paragraph-- 2.5 pages! To help guide the reader, this text should be divided into multiple paragraphs that follow a clear, logical framework.

Suggested breaks:

- End of line 71 seems like a good cut-off for a new paragraph. After this point, you transition into specific US studies and then focus on the “Joo, et al.” and “Lyu and Wehby” publications.
- Line 92 after “short periods of time” seems like a natural break because you stop talking about temporal factors and switch to other factors.

**Line 73:**

In this sentence it is unclear why “longitudinally” is not ideal. Perhaps the authors could consider adding a few words to help out the reader. For example:

“…the assessment was don’t longitudinally, which cannot account for xyz, and included…”

**Lines 74-76:**

You reference two papers for the first time and then refer to them by “Joo, et al.” and “Lyu and Wehby” only later in the paragraph. It would be clearer if you explicitly wrote “Joo, et al.” and “Lyu and Wehby” in this first sentence that introduces their work.

**Lines 84-85 and 89-90:**

The sentence “And while Joo…” is confusing with where it is located. The authors just finished discussing how including US states as a variable in a regression model requires the state effect to remain constant over time, and then the present sentence implies that having a seven-week study observation is a bad thing. This is confusing because the reader may presume (like I did) that having a shorter observation period = less opportunity for the effect to vary over time. Consider appending this sentence to the end of the “Regardless of causes(s)…”. For example:

“…in any seven-week period, such as the seven-week period observed by Lyu and Wehby.”

For further resolution, the authors could consider explicitly contrasting the trade-offs of using longer observational periods in order to include the complexity of periodicity versus using a shorter observational period where the effect is more likely to be constant but periodicity is not captured.

**Lines 101-104:**

These sentences seem out of place because they go back to discussing variability over time. Consider moving to the end of line 92 after “…short periods of time”.

**Lines 121-123:**

The authors saying that they used a modified difference-in-difference approach because of mandates going into effect at different times will likely not be self-explanatory to non-epidemiologists/statisticians. Perhaps a quick explanation as to why difference-in-differences is beneficial could be added at the end of the sentence to help out the reader. For example, a layman’s explanation of how the difference-in-differences approach does not assume exchangeability between the mandate and non-mandate groups. This could also be used to briefly justify not adjusting for the other factors discussed on the previous page (population density, socioeconomic status, political factors, etc.).

**Materials and Methods**

**Lines 132-134:**

Since the information captured by state health departments likely varies by state, it may be beneficial to specify which events qualify for capture/what data sources the OK registry uses (i.e., specify the source population). Does the registry count COVID-19 cases in pharmacies, universities, assisted-care living facilities, etc., or just hospitals?

**Lines 150-153:**

I think “…were calculated for each municipality to establish a mandate” is a typo. It reads as if the presence of a mandate was determined by municipality case density rates, which doesn’t make sense. I’m not sure what this means.

**Additionally:**

Can the authors comment on the number/size of the aggregated non-mandate population? Just so we know there is an adequate non-mandate population size for comparisons with the mandated municipalities (which already have the sizes provided).

**Results:**

**Line 186:**

To support this sentence (i.e., the parallel assumption), perhaps the authors could supplement with a graph.

**Line 189:**

I was confused by this sentence because the preceding paragraph in the Methods and Materials said that there was significant autocorrelation. Specifying that this is for the one-lag moving average term model would improve clarity.

**Lines 190-192:**

- Can the authors comment on the parallel assumption not being violated despite the case rate difference rapidly increasing in the pre-mandate period (i.e., the case rate lines for mandated and non-mandated populations not being parallel since the difference between the two lines increases by 0.32 per unit time)? I had a hard time resolving these two pieces of information, which seem to contradict each other. Is it because the parallel assumption was done prior to date alignment, but the increasing case rate difference in on the aligned time scale?
- Can the authors explain the slope of 0.32 in words since this is the first time they present the slope of mean case rate difference (see comments for **Lines 28-31**)?

**Discussion:**

**Lines 234-236:**

- This sentence is out of place. This concluding sentence (“The fact that…”) is sandwiched by two other sentences (rural versus urban areas and additional possible explanations) that aren’t directly related to it or support it. Instead, this sentence seems to interrupt the discussion about possible reasons for the difference. The authors should consider relocating it.

**Lines 236-243:**

- These sentences are confusing. I think “… ***subsequent*** ***increase*** in case rate difference between communities with mask mandates and those without” indicates there is an increase in the case rate difference after the mandate. This isn’t communicated in the results (which say there is a decrease), so at first I thought I was misinterpreting this sentence. However, the following sentences hypothesize why the mask mandates don’t work as well over time following the mandate (temperature, gradual non-compliance), which makes me think that the “… subsequent increase” sentence is indeed about post-mandate periods of time. Then, the last sentence talks about a spillover effect with mandates benefitting nearby non-mandate regions by reducing transmission to them. I don’t understand how this could explain an ***increase*** in the case rate difference—wouldn’t this transmission reduction decrease the case rate difference?
- I’m not sure if something is missing from the results or this part of the discussion needs work or shuffling to improve clarity. Presently, it is difficult read and understand.

**Line 246:**

Change “compare” to “compared”.

**Line 247:**

Is aligning time to d0 solely responsible for avoiding confounding? Wouldn’t calculating and comparing non-mandate rates with mandate rates on the same calendar date be extremely important for avoiding confounding by temporal factors? I think it is important to mention this, otherwise it reads as if d0 is solely responsible for preventing confounding. In fact, I’m not entirely clear on how d0 avoids temporal confounding when the rate differences for a particular city and the non-mandated population are always calculated on the same day—would it not be this technique of using the same day for the mandated city and non-mandated population comparison that removes confounding and not aligning to d0?

**Lines 270-277**

It is very important for us to remember that observed COVID rates rely on the availability of widespread testing. The authors explain this issue very well and then succinctly describe how/why this bias is limited in their study.

**Lines 280-283:**

It is not explicitly clear why high positivity rate implicate a large number of infections not being detected. Could the authors briefly elaborate?

**Lines 287-300:**

This is an excellently written and technically sound justification for why adjustment for other rate-influencing covariates was not performed.

**Line 313:**

Change “by able” to “be able”.

**Tables and Figures:**

**Table 1:**

I think there is a typo in the rightmost column of Table 1. I think this is supposed to be “Mandate Expiration” and not “Mandate Start”.

**Addition of Figure:**

To improve the reader’s understanding of the results, the authors could consider supplementing an additional figure with:

- Y-axis: mean case rate per 100,000
- X-axis: alignment (d(-45) to d(90))
- One line for case rates in the non-mandated population
- A second line for case rates in the mandated population
- Perhaps mean case rate data points for each population (mandated and non-mandated) at each time point

This will allow the reader to observe the case rates over time for the mandated vs non-mandated groups. Changes in case rates in two populations over time are less abstract and more intuitively understood than changes in case rate differences over time. The possible inclusion of the suggested case rate graph would help non-statistician/epidemiologist readers understand the results.

**Figure 1:**

- Both the regression lines and the data points in Figure 1 are plotted with circles of the same shape. When I first reviewed the figure, I thought the regression lines were also data points. Since the graph is in gray-scale, different shapes/symbols should be used for the regression lines and data points.
- It is not immediately clear what the data points (not lines) are in Figure 1. I think, but am not sure, that each point is the average of the 40 separate differences in case rates between mandated and non-mandated municipalities for each time point. This should be explicitly specified in the Figure title. Better yet, add a legend explaining what the individual data points and regression lines are.
- The methods describe calculating the difference in rates from d(-45) to d(90), but Figure 1 only shows up until d(75) instead of d(90). Is there an error in the axis? If not, perhaps the authors could briefly mention why only up until d(75) is shown.
